# Rheumatoid arthritis and sexuality: A patient survey in France

**DOI:** 10.1186/1471-2474-13-170

**Published:** 2012-09-10

**Authors:** Gisela Kobelt, Brigitte Texier-Richard, Sylvain Mimoun, Anne-Sophie Woronoff, David-Romain Bertholon, Aleth Perdriger, Yves Maugars, Bernard Combe

**Affiliations:** 1 ANDAR, Clermont l’Hérault, France; 2 European Health Economics, 492 chemin des Laurens, 06530 Spéracèdes, Mulhouse, France; 3 EmPatient, Paris, France; 4 Unité d’étude de la sexualité humaine, Hôpital Robert Debré, Paris, France; 5 Centre Hospitalier Universitaire Rennes, Rennes, France; 6 Centre Hospitalier Universitaire Nantes, Nantes, France; 7 Hôpital Lapeyronie, Centre Hospitalier Universitaire Montpellier, Montpellier, France

**Keywords:** Rheumatoid arthritis, Fatigue, Quality of life, Sexuality

## Abstract

**Background:**

The objective of this study was to evaluate the impact of rheumatoid arthritis (RA) on patients’ sexuality and identify disease and other factors such as fatigue that most influence sexual relationships.

**Methods:**

A specific pretested questionnaire was sent to all members of a French patient association (ANDAR). Questions related to demographics, disease status, quality of life (utility, EQ-5D), pain, psychological status (mood), fatigue and emotional and sexual relationships. To isolate the impact of RA, an attempt was made to include a matched sample from the general population.

**Results:**

The analysis included 1271 patients, but only 70 controls agreed to participate and comparisons should therefore be considered with caution. The two groups were similar in terms of age, gender distribution, living conditions and diseases other than RA. However, patients scored worse for global health, mood, fatigue, had a lower utility (0.55 versus 0.65). Controls were more active sexually (69% versus 63%), in particular women (71% versus 60%). Age, gender, living alone, physical function and mood were significant predictors for being sexually active for patients; for controls, age and overall quality of life (utility) were significant predictors.

**Conclusions:**

While it is known that RA has a negative impact on patients’ sexuality, there have been few attempts to quantify the problem. Our study highlights the negative impact of RA on patients’ sexuality, and triggers the question how to include this aspect into care.

## Background

Rheumatoid Arthritis (RA) was one of the first diseases where quality of life (QoL) measurements were included in clinical trials in the mid-eighties [1]. Today investigating patient-reported outcomes has become standard in clinical research and a number of measures are routinely included in trials [2].

Yet, with ever more effective anti-inflammatory and disease modifying treatments available to the treating rheumatologist, the focus in clinical practice is more on control of those objectively measurable disease symptoms such as disease activity targeted by these treatments than on the more subjective and less obvious parts of patients’ well-being. While the relationships between QoL and inflammation, pain and functional limitations have been shown [3-5], control of these symptoms may not fully restore all physical, social and mental aspects of QoL. Studies in France on these topics are rare. The international literature provides an overview of issues such as fatigue, depression, sleep disorder, coping [6-11] and social interactions, family life and sexuality [12-15]. In a survey among French patients around half answered that RA impacted negatively on their family life and/or sexuality [16]. In another observational study in France 80% of patients spontaneously reported fatigue as the most difficult aspect to cope with in daily life, while 15% indicated problems with sexuality [17].

The objectives of the current study were therefore to investigate the impact of RA on patients’ intimate and sexual relationships and identify significant factors such as functional handicap, pain and in particular fatigue that may lead to problems. In order to better isolate problems caused by RA, the study attempted to include a control group from the normal population.

## Methods

### Study approach and subjects

The study was performed as a mail survey addressed to all members of a French patient association (Association Nationale de Défense contre l’Arthrite Rhumatoïde, ANDAR). The control group was enrolled via a second mailing to a random sample of 600 ANDAR members requesting them to ask an acquaintance of similar age and gender to participate. Both groups were adults and provided written consent to participate in the study.

At the time of the study, ethical approval was not required for surveys carried out by patient organisations with their own members, provided informed consent is received from participants. However, the study protocol and questionnaire were submitted to the Commission Nationale d’Information et de Liberté (CNIL) for approval.

### Data

The patient questionnaire was developed by a team of specialists including rheumatologists, a sexologist and patients, and pretested for clarity and relevance in two rounds with two different groups of patients. Questions concerned

demographic data (current): age, gender, family situation, education, professional activity and level of family income

disease data (current, past week): presence of co-morbidity, disease duration, general health status (global visual analogue scale, VAS), functional status (HAQ) [18], disease activity (VAS), pain (VAS) as well as mood (VAS).

utility (current): EQ-5D [19], a generic preference based questionnaire addressing five domains of general well being (mobility, pain, self-care, usual activities, anxiety/depression).

fatigue (past week, past 6 months): VAS as well as a validated French fatigue scale (Pichot) [20] followed by questions on the (self-assessed) causes and the impact of fatigue, coping mechanisms and medical help

sexuality (past 12 months): self-assessed impact of RA, sexual activity and reasons for inactivity, specific difficulties and coping mechanisms, repercussions and medical support

The questionnaire for controls contained the same questions except for disease variables (duration, HAQ, disease activity, pain). For fatigue, only the VAS was used to keep the questionnaire short, and questions relating to possible causes for sexual inactivity (e.g. personal problems, bad health) were modified with the help of a sexologist.

### Analysis

Answers were fully anonymous and returned directly to the data management centre in prepaid envelopes. Thus no verification was possible and questionnaires with missing demographic or disease data or containing inadequate remarks were excluded. We performed basic descriptive analyses on all quantitative and qualitative variables and stepwise logistic and linear multiple regression analyses to isolate factors influencing sexuality and fatigue.

## Results

### Demographics

Within 2 months 1314 patient questionnaires were returned (38%) and 1271 could be included in the analysis. Respondents were from all geographic regions, with a response rate above 30% in all areas; the largest samples were in urban areas (Lyon, Paris) and Southern France. For the control group, response rate was as expected lower (12%) and all 70 responses were included in the analysis. Overall, controls were similar to the patient group in terms of geographic distribution, mean age, gender, education and household income. However, the sample was small and in particular male respondents were few and they appeared to be in poor health. Comparisons should therefore be made with caution and focus more on women.

The characteristics of the two samples are shown in Table 1. The level of education was similar to the French population with ~70% having completed the second cycle (“baccalauréat” or professional diploma) [21] and ~25% with higher education [22]. The proportion of patients working was significantly lower than for controls and the level of professional activity in France. Income distribution was however not significantly different from the general population for both samples [23]. 

**Table 1:** Characteristics of the sample

	**Patients**			**Controls**		
	**Sample**	**Men**	**Women**	**Sample**	**Men**	**Women**
	***N, %, mean***	***N, %, mean***	***N, %, mean***	***N, %, mean***	***N, %, mean***	***N,%, mean***
**N**	**1268**	**200 (16%)**	**1068 (84%)**	**70**	**16 (23%)**	**54 (77%)**
Age (mean)	63.8 (12.4)	66.1	63.3	60.4 (11.7)	63.1	59.6
Living alone	28%	11%*	31%*	24%	31%	25%
**Respondents < 60**	**422 (33%)**	**58 (29%)**	**364 (34%)**	**31(44%)**	**5 (31%)**	**26 (48%)**
Proportion <60 working	55.0%	58.6%	54.4%	74.2%**	60.0%	76.9%
Proportion <60 on full/partial invalidity	45.3%	45.6%	47.8%	-	-	-
**Respondents education**	**1163 (92%)**			**64 (92%)**		
Basic	29.8%			34.4%		
Professional diploma	18.5%			17.2%		
High school	23.7%			21.9%		
University	28.0%			26.6%		
**Respondents household income**	**1183 (93%)**			**64 (92%)**		
<760€	6.1%			9.4%		
760-1800€	39.6%			43.8%		
1800-3000€3	34.1%			20.3%		
>3000€	20.2%			26.6%		
**Respondents sexuality**	**1134 (89%)**	**192 (96%)**	942* (88%)	**70 (100%)**	**16 (100%)**	**54 (100%)**
Sexually active	63%	76%	60%*	69%	63%	71%
<55 years	85%	91%	85%	86%	75%	89%
55-65 years	66%	91%	62%*	76%**	75%	76%
>65 years	36%	57%	32%*	54%**	50%	56%

* p < 0.01 (gender) ** p < 0.01 (controls versus patients).

### Health Data

Health data for both samples are presented in Table 2. The presence of diseases other than RA was similar between patients and controls (61.5% versus 59%), with, however, a difference for women (62% versus 54%).

**Table 2:** Summary disease data

	**Patients**			**Controls**		
	**Sample**	**Men**	**Women**	**Sample**	**Men**	**Women**
	**Mean (SD), %**	**Mean (SD), %**	**Mean (SD), %**	**Mean (SD), %**	**Mean (SD), %**	**Mean (SD), %**
N	1268	200	1068	70	16	54
% with disease other than RA	61.5%	60%	62%	59%	75%	53.7%
Disease duration	19.0 (11.6)	16.6	19.5	-	-	-
HAQ	1.2 (0.8)	0.9	1.3*	-	-	-
Disease activity (VAS)	4.6 (2.2)	4.1	4.7	-	-	-
Pain (VAS)	4.7 (2.2)	4.2	4.7*	-	-	-
Biologic treatment	40.8%	35.4%	42.2%*	-	-	-
Global Health (VAS)	4.5 (1.9)	4.3	4.5	3.8 (2.2)**	3.7	3.8
Fatigue (VAS)	5.6 (2.2)	4.9	5.7	5.3 (2.4)	5.1	5.5
Mood (VAS)	4.2 (2.2)	4.0	4.2	3.9 (2.1)	4.4	3.8
Utility	0.55 (0.3)	0.61	0.54	0.65 (0.3)**	0.57	0.68

*p < 0.01 (gender), **p < 0.01 (controls versus patients).

All VAS = 1(best)-10(worst); Pichot scale = 0(best)-32(worst); HAQ = Health Assessment Questionnaire; SD = standard deviation.

The mean/median disease duration was 19.0/18 (SD11.6) years, but 24% had the diagnosis since less than 10 years. This corresponds well to the age structure in the patient association. Mean and median HAQ was 1.25; 20% of patients had a score above 2, and 56% and 58% used technical aides or medical devices, respectively. Almost all patients were on disease modifying medication (DMARDs), with biologic treatment used more frequently in this sample than the national average estimated at 15% in 2008 [24].

Mean scores on the different VAS (1-10 worst) were generally worse for patients than for controls. Similarly, utility scores for patients (mean/median 0.55/0.66) were lower than those of controls (0.65/0.72) and the general population between 60 and 70 (~0.80) [25]. For patients, the largest impact of RA was seen in the domains pain (95% with moderate/severe pain compared to ~45% in the general population), anxiety/depression (67% with moderate/severe problems compared to ~30%) and usual activities (64% with moderate or severe difficulties compared to ~10%) ^25^. Overall, women had worse disease and worse utility, but judged their overall health better than men.

### Fatigue

Fatigue was an issue for both patients and controls: only 30% of patients and 25% of controls had a score on the VAS of 4 or below (Table 3). Patients with more fatigue were not significantly different in terms of age and disease duration, but appeared to have more severe disease (HAQ, disease activity, pain), lower workforce participation and significantly lower utility. There was a trend for patients at higher education and income levels to have better scores on both fatigue scales. Professionally active patients had less severe disease and thus also less fatigue. A trend for less fatigue was also seen in patients on biologic treatment. On the Pichot fatigue scale (0-32 worst), patients had a mean/median of 17.2/18; 39.4% had scores indicative of a pathological fatigue (>20) while 9% had severe fatigue (>27). In stepwise multiple regression analysis, disease variables (HAQ, disease activity, pain) together with age and mood explained a significant part of fatigue.

**Table 3:** Fatigue

	**Patients**		**Controls**	
	**Fatigue < = 4**	**Fatigue > 4**	**Fatigue < = 4**	**Fatigue > 4**
Respondents 1166	349 (30%)	817 (70%)	17 (25%)	51 (75%)
Age	62.8	64.5	60.1	60.3
Disease duration (years)	18.1	19.5	-	-
Working (%)	24.3%	20.0%	33%	59%
% on Biologics	45.5%	39.9%	-	-
HAQ	0.8	1.4*	-	-
Disease activity	3.1	5.3*	-	-
Global health (VAS)	3.4	4.9*	2.9	4.0
Pain (VAS)	3.1	5.4*	-	-
Mood (VAS)	3.1	4.7*	3.2	4.0
Utility	0.70	0.48*	0.69	0.63

*p < 0.01 high versus low fatigue.

All VAS including fatigue = 1(best)-10(worst); Utility 0(death)-1(full health); HAQ = Health Assessment Questionnaire.

The cause most often cited for fatigue was RA (77.1%) and sleep disturbances (47.4%), and over half of the patients (51.6%) had reduced or stopped leisure activities while 47% felt discouraged. Most patients (59%) coped with fatigue by resting in the afternoon and more than half cited their family as their best support. Three quarters of patients had addressed the issue with their physician and also felt that rheumatologists and physiotherapists were adequately aware of the issue, while employers appeared to lack understanding (60%).

### Sexuality

The majority of patients felt RA to be an obstacle for intimate and sexual relationships (68% and 76%, respectively); for 29% and 33%, respectively, it was a major obstacle. Patients who indicated a strong negative impact of RA were younger but had overall more severe disease (Table 4). Among controls, only 38% (25% of women) felt that they faced obstacles for their sexual relationships.

**Table 4:** Impact of RA on personal and intimate (sexual) relationships

	**Impact of RA**			
	**Emotional relationships**		**Intimate relationships**	
	***Mean***		***Mean***	
	**RA negative**	**RA no effect**	**RA negative**	**RA no effect**
**N**	763	354	834	270
**Age**	61.0*	65.2	60.8*	66.6
**HAQ**	1.3*	1.0	1.3*	0.9
**Fatigue (VAS)**	5.8*	4.9	5.8*	4.9
**Pain (VAS)**	4.7	4.2	4.7	4.2
**Disease Activity (VAS)**	4.9*	3.9	4.8*	3.9
**Mood (VAS)**	4.5*	3.3	4.3*	3.4
**Utility**	0.605*	0.647	0.592*	0.511

*p < 0.01 negative effect/no effect.

All VAS = 1(best)-10(worst); Utility 0(death)-1(full health); HAQ = Health Assessment Questionnaire.

Table 5 summarizes results about sexual activity for both samples and by age groups. Among patients, 63% were sexually active during the past 12 months (60% among women, 76% among men), compared to 69% of controls. For patients not sexually active (N = 425), RA was the main reason (31%) followed by other diseases (8%), while controls (N = 22) indicated personal reasons (73%) and poor health (64%). It is thus not surprising that patients and controls who were sexually active were in better health and had a higher utility. Differences between patients and controls were most striking in women, particularly with advancing age when also the disease has progressed: only 32% of female patients over 65 were active compared to 56% in controls.

**Table 5:** Impact of RA on sexual activity by age group

	**Patients**		**Patients 50-60**		**Patients 60-70**		**Controls**		**Controls 50-60**		**Controls 60-70**	
	***Mean***		***Mean***		***Mean***		***Mean***		***Mean***		***Mean***	
	**Active**	**Not active**	**Active**	**Not active**	**Active**	**Not active**	**Active**	**Not active**	**Active**	**Not active**	**Active**	**Not active**
**N**	712 (62%)	425	226 (78%)	63	240 (60%)	163	48 (69%)	22	15 (75%)	5	15 (71%)	6
**Age**	59.1	67.9*	55.5	56.6	64.5	65.1	57.7	66.2*	53.1	54.8	63.7	63.2
**HAQ**	1.0	1.5*	1.0	1.30*	1.00	1.5*	-	-	-	-	-	-
**Disease Activity**	4.3	5.0*	4.4	4.9	4.1	5.0*	-	-	-	-	-	-
**Pain**	4.3	5.1*	4.4	4.6	4.1	5.0*	-	-	-	-	-	-
**Global health**	4.2	4.7*	3.3	3.6	3.9	4.7	3.3	4.2*	3.6	3.0	3.1	6.0*
**Fatigue**	5.3	6.0*	5.3	5.9*	4.8	6.0*	5.4	5.2	5.4	4.2	5.7	6.5*
**Mood**	3.9	4.6*	4.1	4.7	3.4	4.7*	3.6	4.7*	3.7	3.4	3.0	7.0*
**Utility**	0.61	0.49*	0.59	0.51	0.63	0.52*	0.74	0.45*	0.75	0.38	0.80	0.51*
**Sexual satisfaction**	5.4						5.9					

*p < 0.01 not active/active within group.

All VAS = 1(best)-10(worst); Utility 0(death)-1(full health); HAQ = Health Assessment Questionnaire.

In step-wise logistic regression analysis, higher age, living alone, worse HAQ, lower spirits (mood) and being female had a significant negative impact on whether patients were sexually active. (Table 6) For couples, higher HAQ, higher age and lower spirits (mood) were significant predictors; for patients living alone predictors were age and being a woman. Only one study has identified a similar impact of function, mood and age on sexual activity, although in that study fatigue also was significant [14]. 

**Table 6:** Logistic regression model, sexual activity

	**p<**	**Odds ratio**	**95% C.I. for Odds ratio**	
			**Lower**	**Upper**
Gender (M = 1,F = 0)	,018	1,832	1,109	3,026
Living alone	,000	,276	,187	,408
age	,000	,942	,926	,959
HAQ	,001	,639	,497	,823
mood	,001	1,154	1,062	1,254
Constant	,000	80,654		

Dependent variable: being sexually active. Independent variables tested with stepwise exclusion: age, gender, living alone, education level, income level, professional activity,early retirement due to RA, comorbidity, fatigue, pain, disease activity, HAQ, biologic treatment, mood and utility. Significant effects were found for age, gender, living alone, mood and HAQ: older patients, women, patients living alone, patients with depressed mood and those with a higher HAQ were less likely to be sexually active.

Only 42% of patients indicated that they used devices, drugs or therapy related to their sexual problems. Regardless of whether they were sexually active or not, all cited essentially the same reasons for difficulties with or absence of sexual relationships (absence of desire, vaginal dryness, joint pain) and the same consequences (guilt feelings, frustration, tensions with the partner). Very few patients had discussed the issue with a health professional (18.6%), most of them with either their general practitioners or the gynecologist. Among patients who had not addressed the issue, two thirds did not want to discuss it with a professional and did not feel that they needed support. Controls appeared to find it easier to address the issue with 70% of men and 42% of women having discussed with a health professional.

## Discussion and conclusions

Difficulties in sexual relationships have been mentioned in surveys among patients with RA as an aspect of the disease that is difficult to cope with. However, no study has investigated this in depth in France and the French patient association ANDAR thus decided to perform a specific study on RA patients’ sexuality. Research on fatigue – which is also often mentioned as a very difficult and handicapping aspect of RA - was primarily included because of a hypothesis that fatigue would negatively impact on sexual activity. The results in our study indicate, however, that the impact is not significant. We would argue that the reason for this is the strong impact of physical function (HAQ) which is a significant predictor for having sexual activity or not, but is also correlated with fatigue.

However, with a relatively scattered picture of research on fatigue in RA patients, fatigue is also interesting as a research topic in its own right. Indeed, fatigue was significantly correlated with most disease variables collected in the study: patients with higher fatigue scores had also a higher HAQ, higher disease activity, worse general health, worse pain, more depressed mood and worse quality of life (utility). Among these, HAQ was a strong driver of fatigue, with scores around 3-4 at low HAQ levels to 7 at higher HAQ levels. This confirms the correlation between HAQ and fatigue as reported by patients in an earlier survey [17], but other data on this relationship are scarce. A British study reported similar proportions of patients with fatigue (80%), but found that fatigue reflected pain and signs of depression, not disease activity; functional status was not investigated [9].

An interesting finding was that patients who were working were less fatigued than those working, but also had a lower HAQ. Thus, fatigue and HAQ together have a significant impact on whether a patient can remain in the workforce or not. Contrary to this, controls who were working had significantly more fatigue than those not working. Fatigue may thus be simply a consequence of working for a population in this age group, but one of drivers of being able to work or not for patients.

Previous experience with patient surveys in France indicates answer rates between 30% and 50%. In light of this, and the delicate subject, the overall answer rate of 38% in this survey, with a 90% completion rate for the part sexuality appears excellent and indicative of the importance of this topic to patients. However, the size of the problem is difficult to assess without a comparator group – and such a group is difficult to study, with the limited means of a patient association. We therefore decided to ask a random group of patients to hand the questionnaire to an acquaintance of theirs. The hypothesis that this would provide a sample with similar characteristics as the patients proved correct, although the low number of respondents (12%) was somewhat of a disappointment. Nevertheless, we believe that even the small control group provides insights into the part played by RA in sexual difficulties.

Controls and patients were similar in their demographics, but controls had a surprisingly high number of diseases (59% with an average of 1.1 diseases). This is only slightly less than the co-morbidities of the patients (62% with an average of 1.4 diseases other than RA), and we can assume that this exerts a similar effect in both groups. Significantly fewer patients below 60 (the official retirement age in France) were working than controls, 55% versus 74%, and we would argue that RA is the cause for this difference.

More controls were sexually active, but the difference was small in the population below 55 years. This appears logical, as at that age many patients still have relatively mild RA (mean HAQ 0.97 compared to 1.31 above 55). However, the difference becomes significant at ages above 55: 10-20% fewer patients have sexual activities, with the differences particularly striking for women. As potential issues with age and menopause cannot be different between the two female groups, this difference can thus be considered to be due to RA. Only one other study on sexual activity included a control group [15] and findings were similar.

Few individuals had addressed the issues with a health professional (36% of controls and 18.5% of patients) and those who hadn’t discussed it, were reluctant to do so. However, as more research into this topic is performed, arguments in favor of a more open dialogue between patients and treating physicians might emerge [26].

Our patient sample may, although it is representative of the structure of the patient association, not be entirely representative of the overall RA population in France. Members of patient associations are often older, with longer disease duration and possibly more severe disease, as it takes time before they join. Another issue appears to be the more frequent than average treatment with biologics in the sample. This can likely be partly explained by the longer disease duration and hence advanced disease with patients having failed a number of DMARDs over time. It is also possible that patients on biologics are more aware of issues and more motivated to participate in surveys. A similar picture had emerged in the earlier economic survey performed by ANDAR [17]. This does in our view not diminish the value of the information obtained from the sample.

## Ethical approval

Studies carried out by official patient organizations with their own members do not require approval by ethics committees in France. However, the study has been declared to the French Commission for Information and Liberty (CNIL).

## Competing interests

All authors declare that they have no competing interests.

## Authors contributions

GK has been the lead investigator and author of the manuscript, BTR has been responsible for study administration and in field work, SM has been a member of the scientific committee and has supervised the development of the section on sexuality in the patient questionnaire, ASW has been a member of the scientific committee responsible for patient view and input into the development of the questionnaire, DRB has been responsible for supervision of in-field work, AP has been a member of the scientific committee, YM has been a member of the scientific committee, BC has been a member of the scientific committee, All authors read and approved the final manuscript.

## Pre-publication history

The pre-publication history for this paper can be accessed here:

http://www.biomedcentral.com/1471-2474/13/170/prepub
